# Automatic and Accurate Acquisition of Stem-Related Phenotypes of Mature Soybean Based on Deep Learning and Directed Search Algorithms

**DOI:** 10.3389/fpls.2022.906751

**Published:** 2022-07-11

**Authors:** Yixin Guo, Zhiqiang Gao, Zhanguo Zhang, Yang Li, Zhenbang Hu, Dawei Xin, Qingshan Chen, Rongsheng Zhu

**Affiliations:** ^1^College of Engineering, Northeast Agricultural University, Harbin, China; ^2^College of Arts and Sciences, Northeast Agricultural University, Harbin, China; ^3^College of Agriculture, Northeast Agricultural University, Harbin, China

**Keywords:** soybean phenotype, computer vision, deep learning, directed search algorithms, phenotype acquisition

## Abstract

The stem-related phenotype of mature stage soybean is important in soybean material selection. How to improve on traditional manual methods and obtain the stem-related phenotype of soybean more quickly and accurately is a problem faced by producers. With the development of smart agriculture, many scientists have explored soybean phenotypes and proposed new acquisition methods, but soybean mature stem-related phenotype studies are relatively scarce. In this study, we used a deep learning method within the convolutional neural network to detect mature soybean stem nodes and identified soybean structural features through a novel directed search algorithm. We subsequently obtained the pitch number, internodal length, branch number, branching angle, plant type spatial conformation, plant height, main stem length, and new phenotype-stem curvature. After 300 epochs, we compared the recognition results of various detection algorithms to select the best. Among them, YOLOX had a maximum average accuracy (mAP) of 94.36% for soybean stem nodes and scale markers. Through comparison of the phenotypic information extracted by the directed search algorithm with the manual measurement results, we obtained the Pearson correlation coefficients, R, of plant height, pitch number, internodal length, main stem length, stem curvature, and branching angle, which were 0.9904, 0.9853, 0.9861, 0.9925, 0.9084, and 0.9391, respectively. These results show that our algorithm can be used for robust measurements and counting of soybean phenotype information, which can reduce labor intensity, improve efficiency, and accelerate soybean breeding.

## Introduction

Soybean (*Glycine max* L. Merrill) is one of the most important seed legume crops in the world. It is the main source of edible oil, accounting for nearly 25% of the total global product (Agarwal et al., 2013). The nutritional value of soybean helps prevent heart disease and diabetes to some extent. In addition, soybean is used for both human and animal consumption and is the main type of oil consumption worldwide (Silva et al., 2011). China is the world’s fourth largest soybean producer after the United States, Brazil, and Argentina (Karlekar and Seal, 2020). Since soybean plays an important role in modern economic environments, producing high-quality and high-yield soybean varieties has become the focus of breeding experts.

Wang (1996) indicated that soybean plant type changes in northeast China were mainly reflected in stem enhancement, dwarfing, and branch number reduction and were gradually dominated by fewer branches, a reduction in internodal length, and an increase in main stem node number. Dong (1997) collected data from multiple breeding experts, which indicated that in alpine regions such as Heilongjiang Province, the ideal soybean plant should have the characteristics of high plant height, high node number and density, and low branch number. Simpson and Wilcox (1983) suggested that high yield was positively correlated with late maturity, increased plant height, lodging susceptibility, and grain resistance. The characteristics closely related to seed yield are seed, pod, and node number. The number of nodes is also positively correlated with protein content. Sureshrao et al. (2014) recorded data on 13 yield components to study genetic variability and heritability and analyze genetic progress. The estimated heritability of plant height was higher and showed superior genetic progress. Branch number also has an influence on inheritance. Liu et al. (2011) identified QTLs for six yield-related traits and biological correlations between flowering traits and yield-related traits using simple repeat markers. The proposed yield-related traits included plant height (PH), main stem node number (NNMS), pod number per plant (PNPP), seed number per pod (SNPP), 100-seed weight (SW), and seed yield per plant (SYPP). Xue et al. (2022) suggested that plant height is an important part of plant structure and has an important impact on both crop quality and yield. Thus, mature soybean stem-related phenotypes have become important in soybean material selection. Consequently, developing a rapid, accurate, and high-throughput method to obtain stem-related mature soybean plant phenotypes will improve the breeding process and provide a useful tool for the incorporation of ideal traits into commercial germplasm.

In recent years, the development of intelligent agriculture has led to the expectation that soybean yield prediction, phenotype evaluation, and breeding research will be conducted through deep learning methods. Uzal et al. (2018) introduced a computer vision method that estimates the soybean pod number from the seed number and developed a classic approach based on tailored features extraction (FE), followed by a support vector machine (SVM) classification model, and CNNs. This highlights the particularly high increase in generalization capabilities of a deep learning approach over a classic machine vision approach. Tetila et al. (2020) evaluated five deep learning architectures to classify soybean pest images. Through the evaluation of different fine-tuning and transfer learning strategies for five different deep learning systems, the experimental results show that fine-tuning trained deep learning architecture obtains a higher classification rate than other methods. Maimaitijiang et al. (2020) proposed that multimodal data fusion using low-cost UAV, within a DNN framework, can provide a relatively accurate and robust crop yield estimation and deliver a valuable insight for high-throughput phenotyping and crop field management with high spatial precision. Dos Santos Ferreira et al. (2017) utilized convolutional neural networks (ConvNets or CNNs) in weed detection in soybean crop images and classified weeds into grass and broadleaf, aiming to apply weed-specific herbicide. This study achieved above 98% accuracy, using ConvNets, in broadleaf and grass weed detection in relation to soil and soybean. The average accuracy between all images was above 99%. Moeinizade et al. (2022) developed a robust and automatic approach to estimate the relative maturity of soybean using a time series of UAV images. An end-to-end hybrid model combining convolutional neural network (CNN) and long short-term memory (LSTM) is proposed to extract features and capture the sequential behavior of time-series data. This new information can be used to support plant breeding advancement decisions. Zhou et al. (2021) investigated the potential of estimating flood-induced soybean injuries using UAV-based image features, collected at different flight heights. A deep learning model was used to classify the soybean breeding plots to five FIS ratings, based on the extracted image features. The results indicate that the proposed method is highly promising in estimating FIS for soybean breeding. A soybean flower/seedpod detection system was built to collect growing state data by introducing convolutional neural networks. In this method, observed plant states (e.g., #flowers and #seedpods), in combination with predicted future environmental data, are used to predict soybean crop yields (Pratama et al., 2020). Lu et al. (2022) proposed a soybean yield in-field prediction method based on bean pods and leaf image recognition using a deep learning algorithm, combined with a generalized regression neural network (GRNN).

Although some researchers have explored mature soybean stem-related phenotypes, Li et al. (2021) measured stem length and total soybean main stem length, and Ning et al. (2021) obtained mature soybean stem-related phenotypes, including branch number, main stem, and plant type. It is clear that previous research on soybean plant phenotypes is relatively simple and fragmentary, and phenotypic genomics is based on high-dimensional phenotypic data. The determination of multiple phenotypes, such as pitch number, internodal length, branch number, branching angle, plant type spatial conformation, plant height, main stem length, and stem curvature, has always been problematic for soybean propagation researchers. The problems of manually counting objects are the large variety of characteristics, large numbers, and wide distribution, in addition to the time it takes. In terms of measurement, it is also time-consuming and laborious to measure the length and angle manually using a ruler and angle measuring device. These issues result in difficulty in acquiring mature stem-related soybean phenotypes, ultimately slowing any progress in soybean breeding. Xu et al. (2021) proposed that increasing planting density is an important approach to achieving the potential of soybean yield. Secondly, there was generally a negative correlation between branch number and planting density. Therefore, it is foreseeable that soybeans with few or no branches are currently the focus of research by breeding experts. The algorithm we propose can help breeders to study the phenotype of soybean plants and promote soybean breeding research.

## Materials and Methods

**FIGURE 1:**
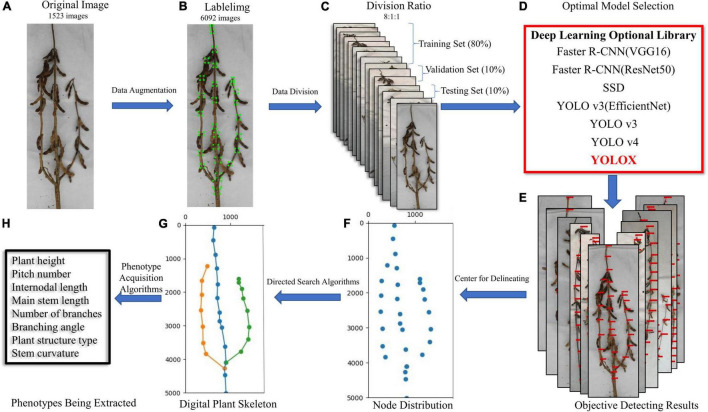
**(A)** Original image, **(B)** labeled image, **(C)** dataset partition, **(D)** optimized model selection, **(E)** object detection results, **(F)** soybean stem node depiction, **(G)** digital plant skeleton rendering, and **(H)** extraction of stem-related phenotypes of soybean plants at maturity. Research flow diagram.

Figure 1 shows an overview of the proposed method. The input of our system includes a series of images of different soybean varieties taken on a platform in a cuboid darkroom (using soybeans planted and cultivated in 2019 and 2021, in fields and pots). The selected images are preprocessed to obtain sufficient data samples. The distributions of the training, validation, and test sets in the dataset are 0.8, 0.1, and 0.1, respectively, and are input into a variety of deep learning networks for training optimization. Through comparison of the test results, the best network was selected as the method for detecting the stem node position of mature soybean. Finally, we mapped the plant type spatial structure and measured the stem-related phenotype of mature soybean plants, via a directed search algorithm.

### Image Acquisition

In this study, soybeans with infinite, finite, and sub-finite podding habits were selected as the experimental subjects. They were then planted at the Northeast Agricultural University experimental base and Xiangyang Farm, in pots and in the field. The field setup was as follows: 2 m × 2 m long rows, plant spacing of 5 cm, and ridge spacing of 55 cm. Three hundred harvested soybean plants were used as experimental samples, and three strains of each variety were selected to extract the soybean phenotype. Potted plants were planted as follows: the same variety was planted in three pots, with a 30-cm space between pots, and 25 plants were harvested as experimental samples. The planting time was mid-May, and harvesting took place in mid-October.

**FIGURE 2:**
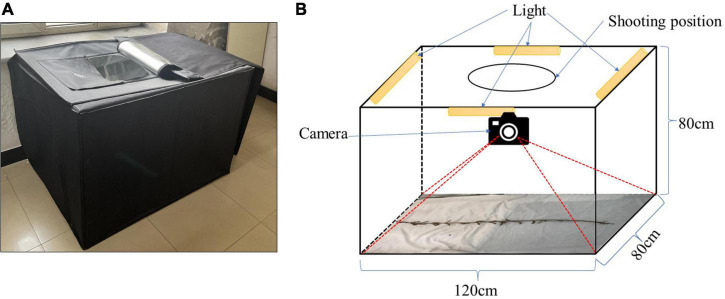
Soybean image collection cuboid darkroom: **(A)** real map and **(B)** structural diagram.

A cuboid darkroom, measuring 120 cm × 80 cm × 80 cm, was used to acquire the RGB images, as shown in Figure 2A. The exterior is constructed of a black synthetic material, the interior contains a silver reflective material, and the cuboid possesses an entrance at the top, which the camera is lowered through. The other sides are closed. Figure 2B details the internal structure of the cuboid darkroom and the arrangement of the soybean plants. Four LED lights are installed on the top four borders at the top of the cuboid darkroom, and reflective materials are arranged around the darkroom to ensure sufficient lighting. An iPhone 13 smartphone and a Canon (DS126291) camera are fitted in a circular shooting port on the top. To prevent the photograph from being affected by background reflections, the background consists of white light-absorbing cloth. When taking an image of a soybean plant, the plant is placed flat on the bottom of the cuboid darkroom and a camera is used to detect it from the top. It is necessary to keep the soybean plants vertical and unobstructed.

The mature soybean stem dataset was set up using JPG format images taken by an iPhone 13 and a Canon (DS126291) camera. The first pair of true leaf expansion points at the bottom of the image is taken as the bottom, and the entire soybean mature plants were photographed as a standard. The shooting lens and background cloth are perpendicular to each other to ensure a clear shot. The image resolution of the iPhone 13 images is 3,024 × 4,032, and that of the Canon (DS126291) camera is 3,456 × 5,184. In addition, the images require a black circular marker with a 1-cm diameter, for use as a scale. In 2021, we obtained 300 field soybeans and 25 potted soybeans, each of which was photographed with a smartphone and a camera from opposite directions, giving four images per soybean. In addition, 223 field soybean images from 2019 were collected, and a total of 1,523 soybean plant images were obtained. The specific soybean plant varieties used are listed in Supplementary Table 1.

At the time of the image acquisition, 100 soybean plants were selected as reference plants to evaluate the algorithm performance. The plant height, pitch number, internodal length, main stem length, stem curvature, branching angle, and other phenotypic information of the reference plants were recorded using a ruler, a protractor, and other tools. All measurements were then recorded in a table.

### Image Preprocessing

**FIGURE 3:**
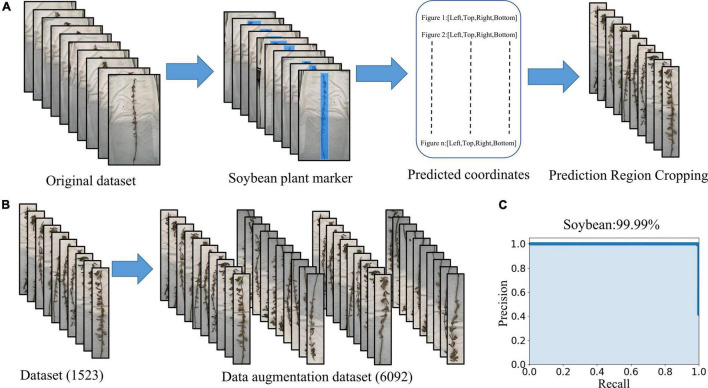
Image preprocessing process: **(A)** remove excess background and keep the smallest bounding rectangle of soybean plants, **(B)** data augmentation, and **(C)** precision–recall curve of soybean plant recognition performance.

LabelImg was used to mark the main part of the mature soybean image. All the images requiring processing were placed into the “JPEGImages” folder and opened through LabelImg. The “create Rectbox” button was used to draw the smallest rectangular boundary to delineate the target object. The label category “soybean” was then applied. The annotation was saved in the specified folder, entitled “Annotations” in “xml” format. We used the YOLOX network to train and test the dataset, and the mAP was 99.99%. The precision–recall curve is shown in Figure 3C and meets the practical application requirements. The object detection network YOLOX identified the entire mature soybean plants by obtaining the coordinates of the upper left and lower right points. The images were clipped using these coordinates, and a soybean mature plant dataset with minimal background information interference is obtained, as shown in Figure 3A. The dataset is available at https://www.kaggle.com/datasets/soberguo/soybeannode.

### Data Augmentation

Training deep learning CNN requires a substantial amount of images to reduce over- and under-fitting, as the original dataset does not include sufficient images for accurate training. Expanding the image set by applying various image augmentation techniques is necessary (Karlekar and Seal, 2020). It is recognized that artificially increasing the number of training samples by applying simple random transformations to input images improves the CNN performance (Chatfield et al., 2014). The augmented image set is created by rotating and flipping the images vertically and horizontally. The augmented image set consists of 6,092 images of which 4,874 (80%) are used for training, 609 (10%) are used for validation, and 609 (10%) are used for testing (Figure 3B). The resulting dataset is relabeled and fed into the deep learning networks we used to detect the training optimization.

### Object Detection

The current object detection algorithm is excellent in agricultural development. Verma et al. (2021) identified insects in soybean crop fields by using YOLO series algorithms in object detection, achieving high accuracy. Zhang et al. (2020) used the improved Faster R-CNN model to detect four tomato leaf diseases: powdery mildew, Fusarium wilt, leaf mold, and ToMV. The model cannot only identify tomato diseases, but also detect the tomato leaf locations. Yuan et al. (2020) detected cherry tomatoes in a greenhouse through a single-shot multi-box detector (SSD). To obtain the best detection effect, we selected a variety of object detection algorithms. These included the typical two-stage object detection algorithm and fast regional convolution neural network (Faster R-CNN) (Ren et al., 2016), ResNet50 and VGG16; backbone networks for training; excellent one-stage object detection algorithms, SSD (Liu et al., 2016), YOLO v3 (Redmon and Farhadi, 2018), YOLO v4 (Bochkovskiy et al., 2020), and YOLOX (Ge et al., 2021). EfficientNet (Tan and Le, 2020) was selected as the YOLO v3 skeleton network. Each model was trained for 300 epochs to allow it to converge. Each object detection model was trained with initialization weights, and the hyperparameters used are presented in Supplementary Table 2. Using our computer hardware solution, CNN was trained on the stem node dataset of soybean mature plants. This is a personal desktop computer with Intel Core i9-10900k CPU, NVIDIA 3080Ti (12G) GPU, and 128 G RAM. We used the desktop to train seven networks in Python language under Windows operating system with the PyTorch framework.

### Directed Search Algorithms

As yet, there is no complete method for obtaining the stem phenotypes of mature soybean plants. Some stem-related phenotypes have been manually obtained in previous studies, but this cannot meet the needs of breeding experts. Therefore, a directed search algorithm is proposed which does not need to study the disassembly and separation of soybean plants or require much human involvement. Consequently, the convenience and accuracy of phenotype acquisition are significantly improved.

**FIGURE 4:**
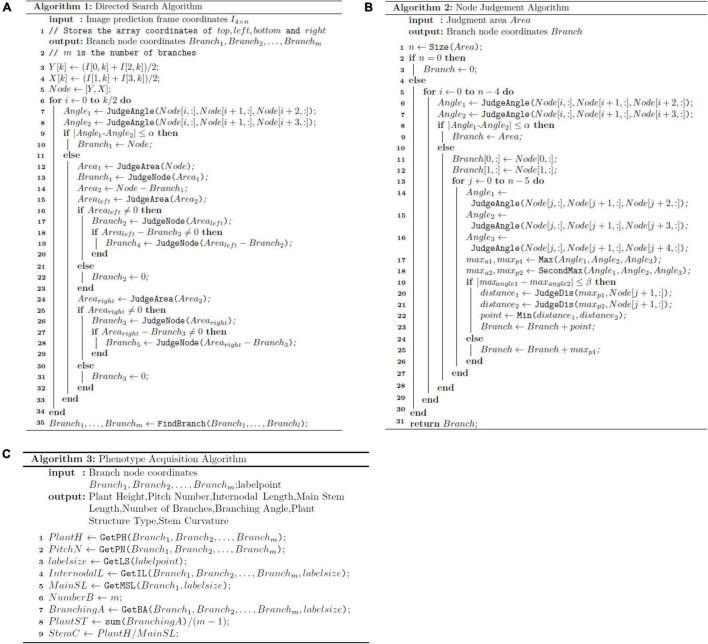
Pseudo-code: **(A)** directed search algorithm, **(B)** node judgment algorithm, and **(C)** phenotype acquisition algorithm.

Algorithm 1 (Figure 4A) was used to draw the plant-type spatial image. The process is as follows: the soybean plant images needed to extract stem-related phenotypes were input into the optimal convolutional neural network, to obtain an array of *n* rows and two columns:


(1) N⁢o⁢d⁢e=[Y,X]

where *X* represents the center point abscissa, *Y* represents the center point ordinate, and *Node* represents the *n*×2 array containing the center points of all the prediction boxes.

Our investigation and analysis of 100 soybean plants in the test set found that the angles formed by the three adjacent stem nodes are all close to 180°, with an average angle of 173.3774 (Supplementary Figure 1). Each branch of the plant is a smooth curve. Under this premise, through the *Node* coordinate array, we can judge whether it is a single-branch or multi-branched soybean plant, according to the angle relationship formed by the three points:


$$A\,n\,g\,l\,e_{1}=\left(N\,o\,d\,e\,\left[i,:\right],N\,o\,d\,e\,\left[i+1,:\right],N\,o\,d\,e\,\left[i+2,:\right]\right),$$

(2)      i=0,1⁢…⁢n-3

$$A\,n\,g\,l\,e_{2}=\left(N\,o\,d\,e\,\left[i,:\right],N\,o\,d\,e\,\left[i+1,:\right],N\,o\,d\,e\,\left[i+3,:\right]\right),$$

(3)      i=0,1⁢…⁢n-3

where *n* represents the length of the *Node* coordinate array and *Angle*_1_ and *Angle*_2_ represent the angles formed by the three points obtained by the cosine theorem. The error angle is set as α. When the difference between *Angle*_1_ and *Angle*_2_ is less than the error angle α, the soybean plant is considered to be a single-branch soybean plant; otherwise, it is considered to be multi-branched.

For single-branch soybean plants, we only need to connect all detected stem nodes according to the y-axis coordinate order to obtain the spatial conformation.

For multi-branched soybean plants, to reduce the detection confusion caused by excessive stem nodes and a decrease in detection accuracy, the algorithm introduces the *JudgeArea* function to delineate a small rectangular area:


(4) l⁢e⁢f⁢t=min⁡(N⁢o⁢d⁢e⁢[0,1],N⁢o⁢d⁢e⁢[-1,1])-p⁢i⁢x⁢e⁢l

(5) t⁢o⁢p=0

(6) r⁢i⁢g⁢h⁢t=max⁡(N⁢o⁢d⁢e⁢[0,1],N⁢o⁢d⁢e⁢[-1,1])+p⁢i⁢x⁢e⁢l

(7) b⁢o⁢t⁢t⁢o⁢m=t⁢h⁢e⁢h⁢e⁢i⁢g⁢h⁢t⁢o⁢f⁢t⁢h⁢e⁢i⁢m⁢a⁢g⁢e

(8) A⁢r⁢e⁢a=(l⁢e⁢f⁢t,t⁢o⁢p,r⁢i⁢g⁢h⁢t,b⁢o⁢t⁢t⁢o⁢m)

where *Area* denotes the circled rectangular area, *left*,*top*,*right*, and *bottom* are the left, top, right, and bottom coordinate values of the rectangular area, respectively, and *pixel* represents the pixel value needed to increase the width of the rectangular area from left to right. We analyzed the data and assessed the pixel value in 5-pixel steps to ascertain the appropriate pixel value, as shown in Supplementary Figure 2. The abscissa represents the pixel value expanded left and right, and the ordinate represents the proportion of soybean plants whose main stem nodes are all within the rectangular area. The analysis showed that when both the left and right are expanded by 30 pixels, all the nodes on the main stem are within the rectangular area; thus, the algorithm adopts the 30-pixel expansion. This is because rectangular areas selected for different soybean plants undergo certain changes; therefore, this variable is used as a variable option that can be changed according to the actual situation.

This function delineates a rectangular area to exclude stem nodes that are not on the main stem, so that we can narrow the scope of the main stem nodes. The stem node coordinates in the rectangular area are stored in the *Node*_1_ array:


(9) $$N\,o\,d\,e_{1}=\left[y_{1},x_{1}\right]$$

Among them, *x_1_* represents the abscissa of the center point, *y_1_* represents the center point ordinate, *Node*_1_ represents an *n*_1_×2 array containing the center points of all the prediction boxes, and *n_1_* is less than *n*.

After selecting the array node, the initial two points are used as the stem nodes on the main stem. We then start from these two points to perform the initial operation and search for the subsequent stem node. If the conditions are met, it is considered a main stem node, and if not, the evaluation of the next stem node is performed until all the stem nodes have been evaluated. Algorithm 2 (Figure 4B) is then introduced to assess the branch stem node. Algorithm 2 (Figure 4B) explains the *JudgeNode* function in Algorithm 1:


$$A\,n\,g\,l\,e_{2\,\_\,1}=\left(N\,o\,d\,e_{1}\,\left[j,:\right],N\,o\,d\,e_{1}\,\left[j+1,:\right],N\,o\,d\,e_{1}\,\left[j+2,:\right]\right),$$

(10) $$\;\;j=0,1\,\ldots \,n_{1}-3$$

$$A\,n\,g\,l\,e_{2\,\_\,2}=\left(N\,o\,d\,e_{1}\,\left[j,:\right],N\,o\,d\,e_{1}\,\left[j+1,:\right],N\,o\,d\,e_{1}\,\left[j+3,:\right]\right),$$

(11) $$\;\;j=0,1\,\ldots \,n_{1}-3$$

$$A\,n\,g\,l\,e_{2\,\_\,3}=\left(N\,o\,d\,e_{1}\,\left[j,:\right],N\,o\,d\,e_{1}\,\left[j+1,:\right],N\,o\,d\,e_{1}\,\left[j+4,:\right]\right),$$

(12) $$\;\;j=0,1\,\ldots \,n_{1}-3$$

(13) $$m\,a\,x_{a\,1},m\,a\,x_{p\,1}=M\,a\,x\,\left(A\,n\,g\,l\,e_{2\,\_\,1},A\,n\,g\,l\,e_{2\,\_\,2},A\,n\,g\,l\,e_{2\,\_\,3}\right)$$

$$max_{a\,2},max_{p\,2}=SecondMax(Angle_{2\,\_\,1},$$

(14) $$\;\;Angle_{2\,\_\,2},Angle_{2\,\_\,3})$$

Among them, *n_1_* represents the coordinate array length of *Node*_1_. *Angle*_2_1_, *Angle*_2_2_, and *Angle*_2_3_, respectively, represent the angle formed by three points obtained by the cosine theorem. *Max* denotes finding the largest angle among the three angles and the corresponding point, and *SecondMax* represents finding the second largest angle among the three angles and the corresponding point. First, the largest angle and the corresponding points *max*_*a*1_ and *max*_*p*1_ are obtained according to the size of the angle, and the largest angle and the corresponding points *max*_*a*2_ and *max*_*p*2_ are also set here. Error angle α is also set here. If the difference between *max*_*a*1_ and *max*_*a*2_ is less than the error angle α, the Euclidean distance from *max*_*p*1_ to *Node*_1_[*j* + 1,:] and from *max*_*p*2_ to *Node*_1_[*j* + 1,:] is calculated through the *JudgeDis* function. The point with the smallest distance is considered the stem node. The algorithm takes the optimum angle value as the primary condition and the optimum distance as the secondary condition, before analyzing all points step by step until the optimal branch route is identified and connecting all the branches with the main stem by the method we propose below.

After the main stem node is ascertained, the stem node arrays on the left and right sides of the main stem are considered through the *JudgeArea* function and examined *via* Algorithm 2 (Figure 4B) until all stem nodes are judged to be complete.

All branches need to be combined following judgment. Here, a method to return branches to the main stem is adopted. This method is based on the lowest point of the branch. The stem node on the main stem with a close distance and a suitable angle is located. When connected with it, the spatial conformation of the multi-branched soybean plants is complete.

After judging all the branches, *m* arrays containing all stem nodes are obtained. By introducing both scale and Algorithm 3, (Figure 4C) we can analyze and measure stem-related phenotypes of mature soybean plants, including plant height, pitch number, internodal length, main stem length, stem curvature, and branching angle:


(15) $$P\,l\,a\,n\,t\,H={\sqrt{\begin{array}{c} {\left(B\,r\,a\,n\,c\,h_{1}\,\left[0,0\right]-B\,r\,a\,n\,c\,h_{1}\,\left[-1,0\right]\right)}^{2}\\ +\left(B\,r\,a\,n\,c\,h_{1}\,\left[0,1\right]-B\,r\,a\,n\,c\,h_{1}\,\left[-1,1\right]\right) \end{array}}}^{2}$$

(16) $$P\,i\,t\,c\,h\,N=l\,e\,n\,\left(B\,r\,a\,n\,c\,h_{j}\right),j=0,1,\ldots ,m$$

$$x={\left(B\,r\,a\,n\,c\,h_{j}\,\left[i,1\right]-B\,r\,a\,n\,c\,h_{j}\,\left[i+1,1\right]\right)}^{2},$$

(17)   ⁢j=0,1,…,m;i=0,1,…,n-1

$$y={\left(B\,r\,a\,n\,c\,h_{j}\,\left[i,0\right]-B\,r\,a\,n\,c\,h_{j}\,\left[i+1,0\right]\right)}^{2},$$

(18)   ⁢j=0,1,…,m;i=0,1,…,n-1

(19) $$I\,n\,t\,e\,r\,n\,o\,d\,a\,l\,L_{i+1}=\frac{\sqrt{x+y}}{l\,a\,b\,e\,l\,s\,i\,z\,e},i=0,1,\ldots ,n-1$$

(20) N⁢u⁢m⁢b⁢e⁢r⁢B=m

$$BranchingA_{j+1}=Angle(Branch_{j}\left[1,:\right],Branch_{j}\left[0,:\right],$$

(21) $$\;\;Branch_{0}\left[1,:\right]),j=0,1,\ldots ,m$$

(22) $$M\,a\,i\,n\,S\,L=\sum_{i=0}^{n-1}I\,n\,t\,e\,r\,n\,o\,d\,a\,l\,L_{i+1},i=0,1,\ldots ,n-1$$

(23) $$S\,t\,e\,m\,C=\frac{P\,l\,a\,n\,t\,H}{M\,a\,i\,n\,S\,L}$$

*PlantH*, *PitchN*, *InternodalL*, *NumberB*, *BranchingA*, *MainSL*, and *StemC* represent the plant height, pitch number, internodal length, main stem length, stem curvature, branching angle, and other related stem phenotypes of mature stage soybean, respectively. *x* represents the square of the difference in the two-point abscissa required to calculate the Euclidean distance. *y* represents the square of the difference between the two-point ordinates necessary to calculate the Euclidean distance. *labelsize* represents the scale.

**FIGURE 5:**
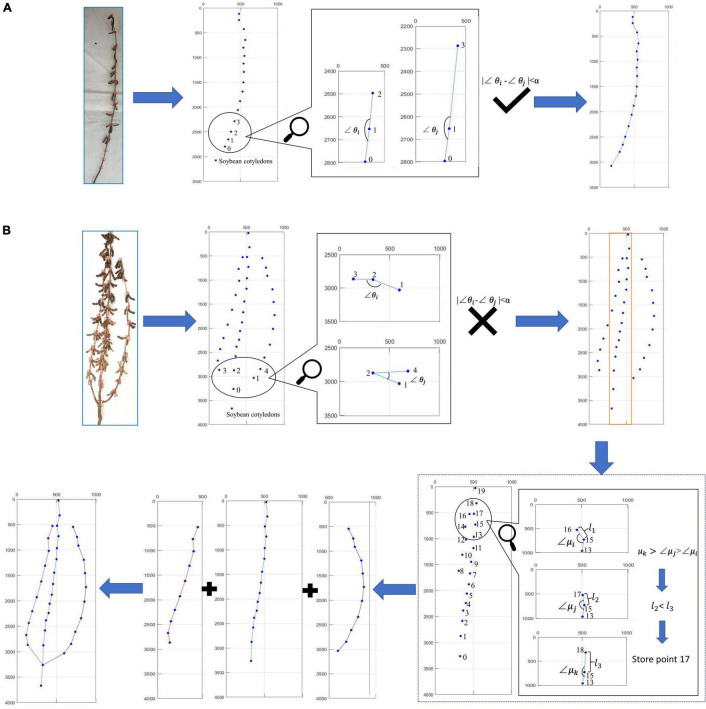
Judgment process of algorithm on actual soybean plant: **(A)** single-branch judgment process and **(B)** multi-branched judgment process.

Figure 5 describes the judging method when the directional search algorithm encounters the actual soybean plants. First, the distinction is made between single-branch and multi-branched soybean plants. The criterion is that all the soybean plant stem nodes can form a smooth curve. Among them, Figure 5A shows the judgment process of single-branch soybean plants, and Figure 5B shows the judgment process of multi-branched soybean plants. If the angle between all adjacent nodes has no large error, the algorithm identifies the soybean as a single-branch soybean plant and draws the soybean plant spatial conformation according to the overall node order. If the angle between adjacent nodes is found to have a large error, the algorithm identifies the soybean as a multi-branched soybean plant and flexibly selects the node sites to be determined, according to the low-end point and top point of the rectangular area. The angle and distance relationships are used to judge the node sites within this area. After the judgment of all the branches is complete, the branch and main stem are combined, according to the returning the branch to the main stem method. Finally, the complete soybean plant spatial conformation is drawn.

### Judgment of Soybean Cotyledon Node

**FIGURE 6:**
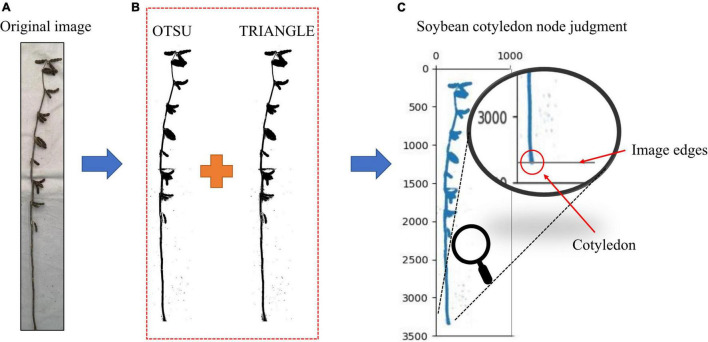
**(A)** Original image, **(B)** Image after binarization, and **(C)** Judgment of soybean cotyledon nodes. Judgment of soybean cotyledon node.

The cotyledonary nodes were not marked when marking the soybean images, as there were certain identification errors that would lead to the selection of the wrong cotyledonary nodes during phenotype calculation. (The accuracy of soybean cotyledon node recognition for the test set samples is shown in Supplementary Table 3). The cotyledonary nodes are highly important for many soybean stem-related phenotypes; therefore, the identification requirements are extremely high. In order to improve the identification accuracy of cotyledonary nodes, we took the cotyledonary nodes as the bottom edge of the image. This method only needs to tangent the soybean plant cotyledonary node to the bottom edge of the image when taking it and convert the original image into a binary image. We used different binary image conversion methods such as OTSU, TRIANGLE, and a combination of the two methods, we tested each method. The results are shown in Supplementary Table 4, the accuracy of the OTSU method is 89.04%, and the accuracy of the TRIANGLE method is 91.44%. The accuracy of the combined method is 98.74%, which is 9.7% and 7.3% higher than that of OTSU and TRIANGLE, respectively. In order to improve the binary image accuracy, we used the Otsu (1979) and TRIANGLE (Zack et al., 1977) thresholds to calculate the binary image, reducing the existing error in the binary image acquisition by calculating the intersection. From the final binary image, the average value of the black pixel coordinates at the bottom edge of the image is obtained as the cotyledon node (Figure 6).

### Evaluation Standard

We evaluated the results from the different networks used in our dataset. For the evaluation, a detected instance was considered a true positive if it had a Jaccard index similarity coefficient, also known as an intersection over union (IOU) (Csurka et al., 2004; He and Garcia, 2009) of 0.5 or more, with a ground-truth instance. The IOU is defined as the ratio of the pixel number in the intersection to the pixel number in the union. The ground-truth instances which did not overlap any detected instance were considered false negatives. The precision, recall, F1 score, AP, and mAP were calculated from these measures (Afonso et al., 2020):


(24) $$P\,r\,e\,c\,i\,s\,i\,o\,n=\frac{T\,P}{T\,P+F\,P}$$

(25) $$R\,e\,c\,a\,l\,l=\frac{T\,P}{T\,P+F\,N}$$

(26) $$F\,1=\frac{2\,P\,r\,e\,c\,i\,s\,i\,o\,n\times R\,e\,c\,a\,l\,l}{P\,r\,e\,c\,i\,s\,i\,o\,n+R\,e\,c\,a\,l\,l}$$

(27) $$A\,P=\sum_{k=1}^{N}P\,r\,e\,c\,i\,s\,i\,o\,n\,\left(k\right)\,△\,R\,e\,c\,a\,l\,l\,\left(k\right)$$

(28) $$m\,A\,P=\frac{\sum_{i}^{M}A\,P_{i}}{M}$$

where *TP* is the number of true positives, *FP* is the number of false positives, *FN* is the number of false negatives, *N* is the total number of images in the test dataset, *M* is the number of classes, *Precision*(*k*) is the precision value at *k* images, and Δ*Recall*(*k*) is the recall change between the *k* and *k-1* images.

Furthermore, the mean absolute error (*MAE*), mean squared error (*MSE*), root mean squared error (*RMSE*), and the correlation coefficient (*R*) were used as the evaluation metrics to assess the counting performance. They take the forms as follows:


(29) $$M\,A\,E=\frac{1}{N}\,\sum_{1}^{N}\left|t_{i}-c_{i}\right|$$

(30) $$M\,S\,E=\frac{1}{N}\,\sum_{1}^{N}{\left(t_{i}-c_{i}\right)}^{2}$$

(31) $$R\,M\,S\,E=\sqrt{\frac{1}{N}\,\sum_{1}^{N}{\left(t_{i}-c_{i}\right)}^{2}}$$

(32) $$R=\sqrt{1-\frac{\sum_{i=1}^{N}{\left(t_{i}-c_{i}\right)}^{2}}{\sum_{i=1}^{N}{\left(t_{i}-\bar{t}\right)}^{2}}}$$

where *N* denotes the number of test images, *t_i_* is the ground-truth count for the *i*th image, *c_i_* is the inferred count for the *i*th image, and $\bar{t}$ is the mean of *t_i_*.

## Results

### Model Training and Evaluation

**FIGURE 7:**
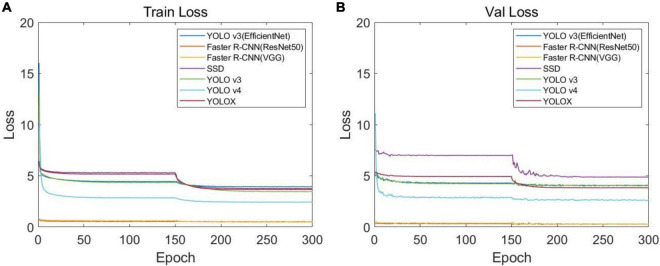
Loss function curves of different networks: **(A)** loss function curve of the training set and **(B)** loss function curve of the verification set.

In order to identify the best CNN model for soybean stem nodes and markers, we trained and evaluated the single-stage object detection algorithms, namely, YOLO v3, YOLO v4, YOLOX, SSD, and YOLO v3 (EfficientNet), and two-stage object detection algorithms, namely, Faster R-CNN (VGG16) and Faster R-CNN (ResNet50). After 300 training rounds, we analyzed the network convergence type. The training loss function curve and verification process are shown in Figure 7. It can be seen that at the beginning of the training stage, the training loss decreased sharply, and after a certain number of iterations, the loss slowly converged to an accurate value.

After the training optimization, we obtained the evaluation indexes of mAP, AP, precision, recall, F1, and frames per second (FPS) on the test set, as shown in Table 1. Among them, “label” represents the identification label of the black circular marker in the target detection network image, and “node” represents the identification label of the soybean plant stem node. By comparing the performance of multiple models, it was found that SSD had the fastest FPS speed, reaching 136.23, but its mAP was only 26.01%, and its detection effect of the stem node was poor. For mAP, YOLOX demonstrated the best performance in all models, reaching 94.37%, but the FPS was much lower than SSD, at only 24.64.

**TABLE 1:** Detection effects of different networks on test sets.

Network name	Category	Precision	Recall	F1	AP	mAP	FPS
Faster R-CNN	Label	60.84%	18.59%	0.28	22.27%	20.94%	12.22
(ResNet50)	Node	25.15%	43.52%	0.32	19.60%		
Faster R-CNN	Label	7.06%	95.73%	0.13	22.25%	24.08%	10.38
(VGG16)	Node	30.37%	47.98%	0.37	25.91%		
SSD	Label	75.00%	0.31%	0.01	21.17%	26.01%	136.23
	Node	66.77%	2.15%	0.04	30.84%		
YOLO v3	Label	83.62%	80.83%	0.82	78.20%	67.53%	14.08
(EfficientNet)	Node	71.04%	51.45%	0.60	56.86%		
YOLO v3	Label	68.28%	66.67%	0.67	47.61%	56.99%	49.27
	Node	76.24%	63.86%	0.70	66.36%		
YOLO v4	Label	93.22%	91.67%	0.92	93.30%	81.70%	28.68
	Node	79.87%	59.87%	0.68	70.09%		
YOLOX	Label	96.90%	96.15%	0.97	96.15%	94.37%	24.64
	Node	93.56%	92.53%	0.93	92.58%		

**FIGURE 8:**
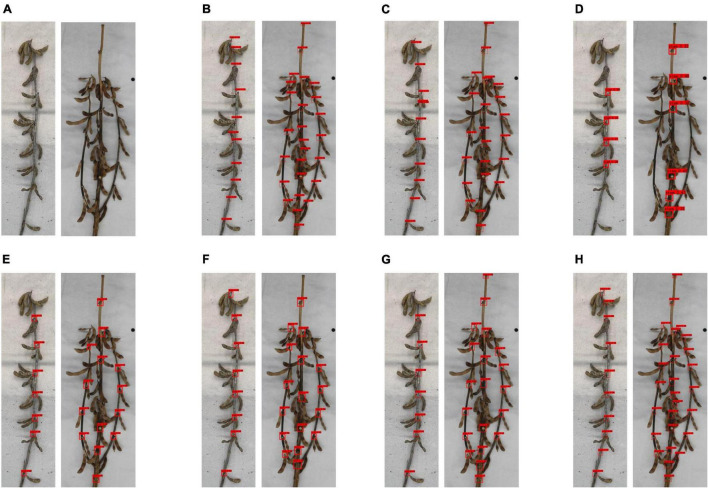
Prediction effect of different networks on soybean plants: **(A)** original image, **(B)** detection results of Faster R-CNN (ResNet50), **(C)** detection results of Faster R-CNN (VGG16), **(D)** detection results of SSD network, **(E)** detection results of YOLO v3 (EfficientNet) network, **(F)** detection results of YOLO v3 network, **(G)** detection results of YOLO v4 network, and **(H)** detection results of YOLOX network.

In addition, to intuitively show the different prediction effects of various network models, we selected single-branch and multi-branched soybean plants as samples, as shown in Figure 8. It was found that SSD had a worse prediction effect than other networks, and some obvious node information was not clearly identified. Faster R-CNN (VGG16) and Faster R-CNN (ResNet50) have a poor judgment of unobvious node information on the stem, and misjudgment is possible. YOLO v3, YOLO v4, and YOLO v3 (EfficientNet) had a poor soybean plant top node recognition effect, often missing the top node. YOLOX had the best detection effect of all network models and is superior to other networks in judging whether the node information is obvious on the stem or the top node information.

Considering that our phenotype research focuses on average accuracy, we adopted YOLOX as the network to identify node and scale labels.

### Phenotypic Identification Results

The optimal object detection network YOLOX was used to identify mature soybean images, and the mature soybean plant stem-related phenotypes were measured and counted by a directed search algorithm.

**FIGURE 9:**
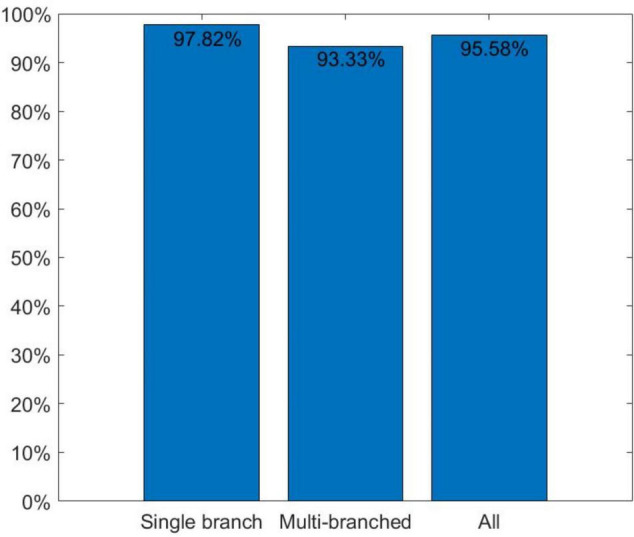
Branch judgment accuracy.

First, we analyzed the algorithm branch judgment results and created the histogram shown in Figure 9. It was found that our algorithm had an accuracy rate of 97.82% for single-branch soybean plants and 93.33% for multi-branched soybean plants, which is slightly weaker than the detection effect of single-branch soybean plants. The overall accuracy rate reached 95.58%, which is adequate for the needs of daily breeding specialists.

The phenotypic information of 100 soybean plants, manually recorded during the acquisition period, was compared with the proposed algorithm to draw the correlation analysis diagram, shown in the figure, to evaluate the reliability and stability of the proposed algorithm. The correlation between the manual measurement results and algorithm measurement results of 100 selected soybeans was analyzed. Subsequently, a scatter plot and a regression line were drawn.

**FIGURE 10:**
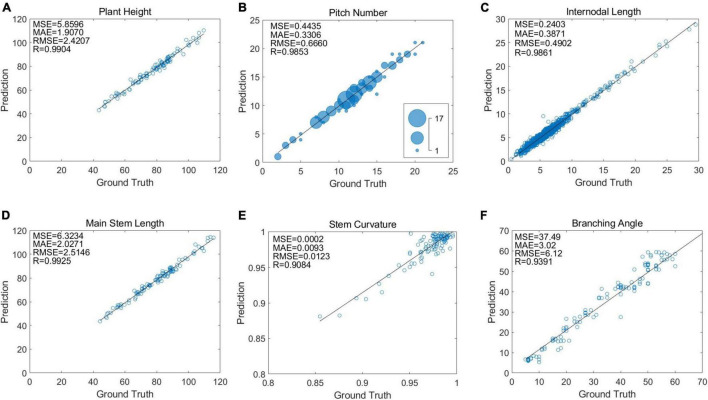
Correlation analysis between true value and predicted value of soybean plant phenotypic information: **(A)** the correlation analysis diagram between the true value and the predicted value of plant height, **(B)** the correlation analysis diagram between the true value and the predicted value of pitch number, **(C)** the correlation analysis diagram between the true value and the predicted value of internodal length, **(D)** the correlation analysis diagram between the true value and the predicted value of main stem length, **(E)** the correlation analysis diagram between the true value and the predicted value of stem curvature, and **(F)** the correlation analysis diagram between the true value and the predicted value of branching angle.

Figure 10A shows the correlation analysis between the soybean’s actual plant height and that predicted by the algorithm. The soybean plant height was calculated as the vertical distance from the top stem pixel to the cotyledon node baseline. One hundred soybeans were selected as materials to evaluate the true and predicted soybean plant height values. By creating the scatter plot and evaluating the correlation, the Pearson correlation coefficient R of the plant height was 0.9904 and the average absolute error was 1.907 cm. From the randomly selected 100 soybean plants, the plant height ranged from 40 to 120 cm, of which heights of 80–100 cm were the majority.

Figure 10B shows the actual and predicted node correlation analysis of the 100 selected soybeans. Each soybean plant node refers to the stem between two adjacent nodes. The Pearson correlation coefficient R for the node number of each soybean was 0.9853, and the average absolute error was 0.3306. In addition, the bubble size represents the number of repetitions between the true and predicted values. For the randomly selected soybean plants, it was found that most of the soybean plant nodes were within 10–15 nodes, and generally, fewer nodes exist in the branches of multi-branched soybeans.

Figure 10C shows the actual and predicted internode spacing correlation analysis of the 100 selected soybeans. The internode spacing refers to the actual length of each internode. A total of 1,438 internode spacings were obtained from 100 soybean plants, the correlation of which was assessed. The internode spacing Pearson correlation coefficient was 0.9861, and the average absolute error was 0.3871 cm. In addition, it is worth noting that the vast majority of internodes fall within the range of 0–10 cm, and larger internodes are uncommon.

Figure 10D shows the actual and predicted stem length correlation analysis of the 100 selected soybean plants. The entire length of the main stem was determined by accumulating the length of all stems and nodes in turn. The Pearson correlation coefficient was 0.9925, and the average absolute error was 2.0271 cm.

Figure 10E shows the correlation analysis between the actual and predicted stem curvature values of the 100 selected soybean plants. The stem curvature is based on the main stem and is represented by the plant height-to-main stem length ratio. The Pearson correlation coefficient was 0.9084. For sampling, the 100 soybean plants were generally in a near-upright state, with only a few bent plants, indicating that this time soybean plants selected should have a certain degree of lodging resistance.

Figure 10F shows the correlation analysis of the actual and predicted branching angle values of the selected multi-branch soybeans. The branching angle is defined as the natural angle between the growth direction of the lower branch and the main stem. The Pearson correlation coefficient was 0.9391, and the average absolute error was 3.02°.

**FIGURE 11:**
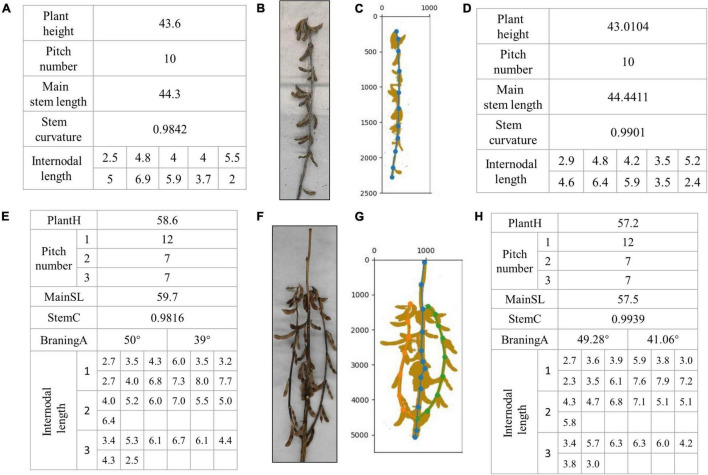
Results of phenotype calculation and algorithm prediction of single-branch soybean plants and multi-branched soybean plants: **(A–D)** are branching-free soybean plants, and **(E–H)** are multi-branched soybean plants.

Figure 11 shows the artificial phenotype calculation results and algorithm predictions of the single-branch and multi-branched soybean plants. Figures 11A–D features single-branch soybean plants. Figure 11B is an original image taken by us. Figure 11C is the soybean plants’ spatial image drawn by the algorithm. Figure 11A shows the actual stem-related phenotype values of single-branch soybean plants. Figure 11D shows the predicted stem-related phenotype values of single-branch soybean plants. It was found that the single-branch soybean plant drawing process is relatively simple and does not require a complex judgment process. The node position can be accurately identified, and the phenotypic information can also be precisely obtained. Figures 11E–H feature the multi-branched soybean plants. Figure 11F is our original image, and Figure 11G shows the soybean plant spatial image plants drawn by the algorithm. For multi-branched soybean, the algorithm initially judges the main stem and displays its phenotypic information. The error between the predicted value and the actual value is very small, which meets the needs of automatic calculation. In addition, the extraction of the multi-branched soybean branch phenotype is complex and includes the branch angle, node number of each branch, and node spacing. The branch detection algorithm can still accurately judge the branch and describe the corresponding phenotypic information, of multi-branch soybeans.

### Algorithm Computational Performance

**FIGURE 12:**
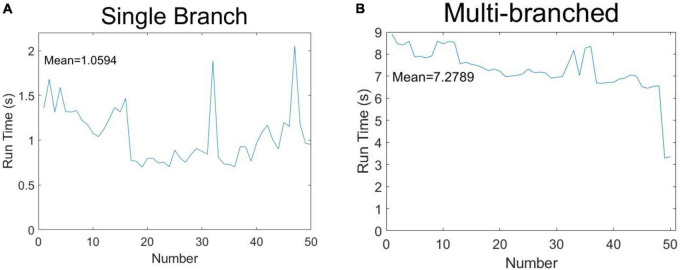
Speculation time of soybean phenotype acquisition: **(A)** single-branch soybean plants and **(B)** multi-branched soybean plants.

In order to demonstrate the algorithm performance in terms of identifying soybean stem-related phenotypes, 50 soybeans were selected for running time measurement according to the classification of single-branch and multi-branched soybeans. The results are shown in Figure 12. Figure 12A shows the phenotypic calculation running time of each single-branch soybean plant, which was an average of 1.0594 s. Figure 12B shows the phenotypic calculation running time for each multi-branched soybean plant, which averaged 7.2789 s. Overall, the calculation time of each soybean phenotype was about 4.1692 s, far less than the artificial phenotype calculation time.

## Discussion

### Plant Branching Morphology

**FIGURE 13:**
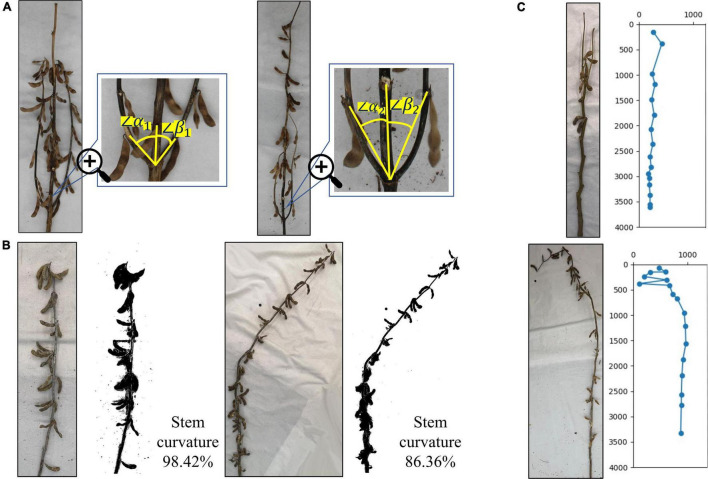
Identification of branch morphology, stem curvature, and specific plants: **(A)** difference in branch morphology of plants, **(B)** difference in stem curvature, and **(C)** identification of specific plants.

For soybean plants with complex branches, plant branch morphology is an important soybean plant phenotype and is mainly dependent on the angle between each branch and the main stem, that is, the convergence degree between the lower branch and the main stem at maturity. As shown in the figure, the natural angles between the growth direction of the lower branch and the main stem are α and β. When the average value of all angles is less than 30°, it is convergent, and when the angle is greater than 30°, but less than 60°, it is semi-open. When the angle is greater than 60°, it is open. The soybean plant branch is mainly composed of convergent and semi-open types. Its defining characteristic is that the branch slowly extends upward from the main stem, close to the main stem growth. The figure on the left side of Figure 13A shows a semi-open type, as the average angle between the left and right branches and the main stem is greater than 30°, but less than 60°. The figure on the right side of Figure 13A shows a convergent example, as the average angle between the left and right branches and the main stem is less than 30°.

### Stem Curvature

The soybean plant stem curvature is the redefined soybean plant phenotype, consisting of the ratio of the true soybean height to the main stem length. The value range is 0–1. The closer the curvature is to 0, the more curved the soybean plant is, and the closer the curvature is to 1, the more erect the soybean plant is. This reflects the overall bending degree of the soybean plant and highlights its lodging resistance. Figure 13B illustrates that the actual curvature of branchless soybean plants on the left side is 98.42%, and the bending degree is very low, which is close to the upright plant and has strong lodging resistance. The actual curvature of the non-branched soybean plant, shown on the right side of Figure 13B, was 86.36%. The soybean plant began to slowly bend from the nodes below the middle, away from its original growth trajectory, and had poor lodging resistance.

### Identification of Specific Plants

Soybean growing environments are changing, along with their growth methods, resulting in mature soybean plants having a variety of morphological characteristics. There are some special forms. Figure 13C shows the special soybean plants produced during the soybean growth process. The main specificity is reflected at the start of the bifurcation at the top of the plant and the formation of two top points. This causes errors in the algorithm’s automatic identification, leading to ignoring some sections and calculating the interval of others. However, generally, the situation can be resolved and the true soybean plant state can be identified, allowing the true phenotype to be fully extracted. In addition, the soybean plant morphology is changed not only by its environment, but also by human influence. Figure 13C shows the damage to the top of the soybean plant from humans. The whole soybean in the damaged part is broken, causing it to grow curved and downward. In the algorithm automatic identification process, phenotypic information such as node position and spacing can still be correctly identified before the damaged section. However, after it, there may be chaotic judgments, and the next node position information will not be correct.

### Choice of Error Angle

**FIGURE 14:**
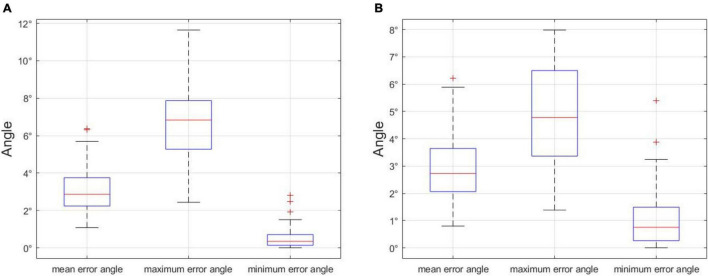
**(A)** Error angle analysis of the whole stem node of soybean plant, and **(B)** error angle analysis of stem node of the lower half of soybean plant. Statistical analysis of error angles.

In order to choose an appropriate error angle to distinguish unbranched soybeans from multi-branched, we selected 500 soybean plant images in the dataset, including unbranched and multi-branched plants, the maximum error angle, minimum error angle, and average error angle of 500 soybean plants were counted, and the process was repeated 100 times. The analysis of the error angle is shown in Figure 14A, the *x*-axis denotes the minimum error angle, the average error angle, and the maximum error angle, and the *y*-axis denotes the angle value of the error angle. In 100 repeated experiments, we got the upper limit of the maximum error angle of 11.6428°. In addition, since the curved part of the soybean plant generally appears at the top and the branched part generally appears at the bottom, we divided the soybean plant into the upper and lower halves of the stem node, according to the principle of equal division. The analysis of the error angle of the lower half of the stem node (Figure 14B) shows that the upper limit of the maximum error angle is 7.8530° in 100 repeated experiments. If the branch judging is based on the overall error angle of the soybean plant, selecting a value slightly larger than 11.6428° is recommended, and if it is based on the lower half of the stem node error angle, it is recommended that a value slightly larger than 7.8530° is selected. In this paper, we opted to judge the branch according to the error angle of the lower half of the stem node and selected an error angle of 10°.

### Digital Plant Skeleton

Through deep learning and our directional search algorithm, we obtained the stem-related phenotypes of soybean plants with high throughput and accuracy and plotted the spatial conformation of soybean plants. We revealed the topological structure of soybeans, initially using digital plants, followed by the analysis and judgment of real soybean pods. In the next study, we will further examine the pods of non-decomposed soybean plants, obtain their corresponding phenotypes, grain number, length, and width, and return the obtained soybean pods to the node we detected. Consequently, we will obtain the spatial conformation of soybean plants containing pods. In addition to the soybean stem-related phenotypes that have already been, we will also obtain the pods per plant, pods per node, pod grains per node, pod length, pod width, and other soybean-related phenotypes which fully show soybean plant topological structure and phenotypic information.

## Conclusion

In this paper, a method for automatically calculating the stem-related phenotypes of whole soybean plants, based on deep learning and a directed search algorithm, is proposed. This method detected the required node position information characteristics based on deep learning and used a directed search algorithm to extract the soybean stem-related phenotypes.

A soybean plant dataset, composed of 6,092 images, was established. The images were taken from several soybean varieties grown in various environments and were preprocessed. Through identifying and comparing datasets, we found that the overall performance of the YOLOX network was the best, and the mAP of the test set was 94.37%. In addition, the Pearson correlation coefficients R of plant height, pitch number, internodal length, main stem length, stem curvature, and branching angle were 0.9904, 0.9853, 0.9861, 0.9925, 0.9084, and 0.9391, respectively. The average running time was 4.1629 s, which is much less than the manual operation time, meeting automatic calculation requirements and verifying our method as both efficient and convenient.

## Data Availability Statement

The original contributions presented in this study are included in the article/Supplementary Material, further inquiries can be directed to the corresponding authors.

## Author Contributions

YG: formal analysis, investigation, methodology, visualization, and writing—original draft. ZG: supervision and validation. YL, ZH, and ZZ: project administration and resources. DX: writing—review and editing and funding acquisition. QC: writing—review and editing, funding acquisition, and resources. RZ: designed the research of the article, conceptualization, data curation, funding acquisition, resources, and writing—review and editing. All authors agreed to be accountable for all aspects of their work to ensure that the questions related to the accuracy or integrity of any part is appropriately investigated and resolved, and approved for the final version to be published.

## Conflict of Interest

The authors declare that the research was conducted in the absence of any commercial or financial relationships that could be construed as a potential conflict of interest.

## Publisher’s Note

All claims expressed in this article are solely those of the authors and do not necessarily represent those of their affiliated organizations, or those of the publisher, the editors and the reviewers. Any product that may be evaluated in this article, or claim that may be made by its manufacturer, is not guaranteed or endorsed by the publisher.

## Supplementary Material

Supplementary Figure 1 Angle analysis of three adjacent stem nodes on branches of soybean plants.

Supplementary Figure 2 Rectangular selection area extended pixel selection.
