# Supplementary material for: Automatic and Accurate Acquisition of Stem-Related Phenotypes of Mature Soybean Based on Deep Learning and Directed Search Algorithms
Source: Front Plant Sci. 2022 Jul 11;13:906751. doi: 10.3389/fpls.2022.906751 (PMC9310015; doi:10.3389/fpls.2022.906751)
Supplement: Supplementary file 1 [file Table_1.DOCX]

**Table S1**. Specific information about the selected soy material

| Variety or number | Planting quantity | Planting site | Planting method |
| --- | --- | --- | --- |
| 6927 | 3 | Xiangyang Farm | field |
| 6612 | 3 | Xiangyang Farm | field |
| 6901 | 3 | Xiangyang Farm | field |
| 6742 | 3 | Xiangyang Farm | field |
| 6890 | 3 | Xiangyang Farm | field |
| 6631 | 3 | Xiangyang Farm | field |
| 6804 | 3 | Xiangyang Farm | field |
| 6795 | 3 | Xiangyang Farm | field |
| 6601 | 3 | Xiangyang Farm | field |
| 6603 | 3 | Xiangyang Farm | field |
| 6799 | 3 | Xiangyang Farm | field |
| 6774 | 3 | Xiangyang Farm | field |
| 6797 | 3 | Xiangyang Farm | field |
| 6900 | 3 | Xiangyang Farm | field |
| 6796 | 3 | Xiangyang Farm | field |
| 6777 | 3 | Xiangyang Farm | field |
| C7 | 3 | Xiangyang Farm | field |
| 6891 | 3 | Xiangyang Farm | field |
| 6876 | 3 | Xiangyang Farm | field |
| 6875 | 3 | Xiangyang Farm | field |
| 6882 | 3 | Xiangyang Farm | field |
| 6885 | 3 | Xiangyang Farm | field |
| 6931 | 3 | Xiangyang Farm | field |
| 6888 | 3 | Xiangyang Farm | field |
| 6817 | 3 | Xiangyang Farm | field |
| 6936 | 3 | Xiangyang Farm | field |
| Cx26 | 3 | Xiangyang Farm | field |
| Cx175 | 3 | Xiangyang Farm | field |
| Cx168 | 3 | Xiangyang Farm | field |
| Cx158 | 3 | Xiangyang Farm | field |
| Cx179 | 3 | Xiangyang Farm | field |
| Cx178 | 3 | Xiangyang Farm | field |
| Cx142 | 3 | Xiangyang Farm | field |
| Cx227 | 3 | Xiangyang Farm | field |
| Cx124 | 3 | Xiangyang Farm | field |
| Cx74 | 3 | Xiangyang Farm | field |
| Cx318 | 3 | Xiangyang Farm | field |
| Cx317 | 3 | Xiangyang Farm | field |
| Cx319 | 3 | Xiangyang Farm | field |
| Cx240 | 3 | Xiangyang Farm | field |
| Cx320 | 3 | Xiangyang Farm | field |
| Cx324 | 3 | Xiangyang Farm | field |
| 6894 | 3 | Xiangyang Farm | field |
| 6736 | 3 | Xiangyang Farm | field |
| 6896 | 3 | Xiangyang Farm | field |
| 6656 | 3 | Xiangyang Farm | field |
| 6686 | 3 | Xiangyang Farm | field |
| 6643 | 3 | Xiangyang Farm | field |
| Cx61 | 3 | Xiangyang Farm | field |
| Cx49 | 3 | Xiangyang Farm | field |
| 6621 | 3 | Xiangyang Farm | field |
| 6622 | 3 | Xiangyang Farm | field |
| 6604 | 3 | Xiangyang Farm | field |
| 6768 | 3 | Xiangyang Farm | field |
| 6791 | 3 | Xiangyang Farm | field |
| Cx250 | 3 | Xiangyang Farm | field |
| Cx290 | 3 | Xiangyang Farm | field |
| Cx248 | 3 | Xiangyang Farm | field |
| Cx257 | 3 | Xiangyang Farm | field |
| Cx249 | 3 | Xiangyang Farm | field |
| Cx260 | 3 | Xiangyang Farm | field |
| Cx253 | 3 | Xiangyang Farm | field |
| Cx56 | 3 | Xiangyang Farm | field |
| Cx145 | 3 | Xiangyang Farm | field |
| Cx91 | 3 | Xiangyang Farm | field |
| Cx30 | 3 | Xiangyang Farm | field |
| Cx32 | 3 | Xiangyang Farm | field |
| Cx162 | 3 | Xiangyang Farm | field |
| Cx36 | 3 | Xiangyang Farm | field |
| Cx256 | 3 | Xiangyang Farm | field |
| Cx172 | 3 | Xiangyang Farm | field |
| Cx275 | 3 | Xiangyang Farm | field |
| Cx273 | 3 | Xiangyang Farm | field |
| Cx29 | 3 | Xiangyang Farm | field |
| Cx164 | 3 | Xiangyang Farm | field |
| Cx196 | 3 | Xiangyang Farm | field |
| Cx143 | 3 | Xiangyang Farm | field |
| 6629 | 3 | Xiangyang Farm | field |
| 6986 | 3 | Xiangyang Farm | field |
| 6613 | 3 | Xiangyang Farm | field |
| 6636 | 3 | Xiangyang Farm | field |
| 6625 | 3 | Xiangyang Farm | field |
| 6834 | 3 | Xiangyang Farm | field |
| 6776 | 3 | Xiangyang Farm | field |
| 6984 | 3 | Xiangyang Farm | field |
| 6850 | 3 | Xiangyang Farm | field |
| 6992 | 3 | Xiangyang Farm | field |
| 6701 | 3 | Xiangyang Farm | field |
| 6986 | 3 | Xiangyang Farm | field |
| 6989 | 3 | Xiangyang Farm | field |
| 6712 | 3 | Xiangyang Farm | field |
| 6741 | 3 | Xiangyang Farm | field |
| 6981 | 3 | Xiangyang Farm | field |
| 6965 | 3 | Xiangyang Farm | field |
| 6993 | 3 | Xiangyang Farm | field |
| 6727 | 3 | Xiangyang Farm | field |
| 6728 | 3 | Xiangyang Farm | field |
| 6978 | 3 | Xiangyang Farm | field |
| Cx272 | 3 | Xiangyang Farm | field |
| Cx262 | 3 | Xiangyang Farm | field |
| Flat stem soybean | 1 | NEAU Experimental Base | potted plants |
| DN252 | 3 | NEAU Experimental Base | potted plants |
| HENONG60 | 1 | NEAU Experimental Base | potted plants |
| HENONG76 | 2 | NEAU Experimental Base | potted plants |
| HENONG91 | 3 | NEAU Experimental Base | potted plants |
| HEINONG48 | 3 | NEAU Experimental Base | potted plants |
| HEINONG51 | 2 | NEAU Experimental Base | potted plants |
| QINONG5 | 7 | NEAU Experimental Base | potted plants |
| SUINONG52 | 3 | NEAU Experimental Base | potted plants |
