# Supplementary material for: Automatic and Accurate Acquisition of Stem-Related Phenotypes of Mature Soybean Based on Deep Learning and Directed Search Algorithms
Source: Front Plant Sci. 2022 Jul 11;13:906751. doi: 10.3389/fpls.2022.906751 (PMC9310015; doi:10.3389/fpls.2022.906751)
Supplement: Supplementary file 2 [file Table_2.DOCX]

**Table S2**. Hyperparameter selection for different networks

| Network name | Learning rate | Batch size | optimizer |
| --- | --- | --- | --- |
| Faster R-CNN (ResNet50) | 0.001 | 4 | Adam |
| Faster R-CNN (VGG16) | 0.001 | 4 | Adam |
| SSD | 0.0005 | 4 | Adam |
| YOLO v3 (EfficientNet) | 0.001 | 4 | Adam |
| YOLO v3 | 0.001 | 4 | Adam |
| YOLO v4 | 0.0001 | 4 | Adam |
| YOLOX | 0.0001 | 4 | Adam |
