# Supplementary material for: Automatic and Accurate Acquisition of Stem-Related Phenotypes of Mature Soybean Based on Deep Learning and Directed Search Algorithms
Source: Front Plant Sci. 2022 Jul 11;13:906751. doi: 10.3389/fpls.2022.906751 (PMC9310015; doi:10.3389/fpls.2022.906751)
Supplement: Supplementary file 3 [file Table_3.DOCX]

**Table S3**. Detection effect of different networks on soybean cotyledon nodes

| Network name | Accuracy |
| --- | --- |
| Faster R-CNN (ResNet50) | 56.98% |
| Faster R-CNN (VGG16) | 61.74% |
| SSD | 15.11% |
| YOLO v3 (EfficientNet) | 28.74% |
| YOLO v3 | 47.13% |
| YOLO v4 | 41.70% |
| YOLOX | 73.89% |
