# Supplementary material for: Automatic and Accurate Acquisition of Stem-Related Phenotypes of Mature Soybean Based on Deep Learning and Directed Search Algorithms
Source: Front Plant Sci. 2022 Jul 11;13:906751. doi: 10.3389/fpls.2022.906751 (PMC9310015; doi:10.3389/fpls.2022.906751)
Supplement: Supplementary file 4 [file Table_4.DOCX]

| Network name | Accuracy | Error rate |
| --- | --- | --- |
| OTSU | 89.04% | 10.96% |
| TRIANGLE | 91.44% | 8.56% |
| OTSU and TRIANGLE | 98.74% | 1.26% |

**Table S4**. Accuracy of different image binarization methods
