# Supplementary figures and images for: Automatic and Accurate Acquisition of Stem-Related Phenotypes of Mature Soybean Based on Deep Learning and Directed Search Algorithms
Source: Front Plant Sci. 2022 Jul 11;13:906751. doi: 10.3389/fpls.2022.906751 (PMC9310015; doi:10.3389/fpls.2022.906751)

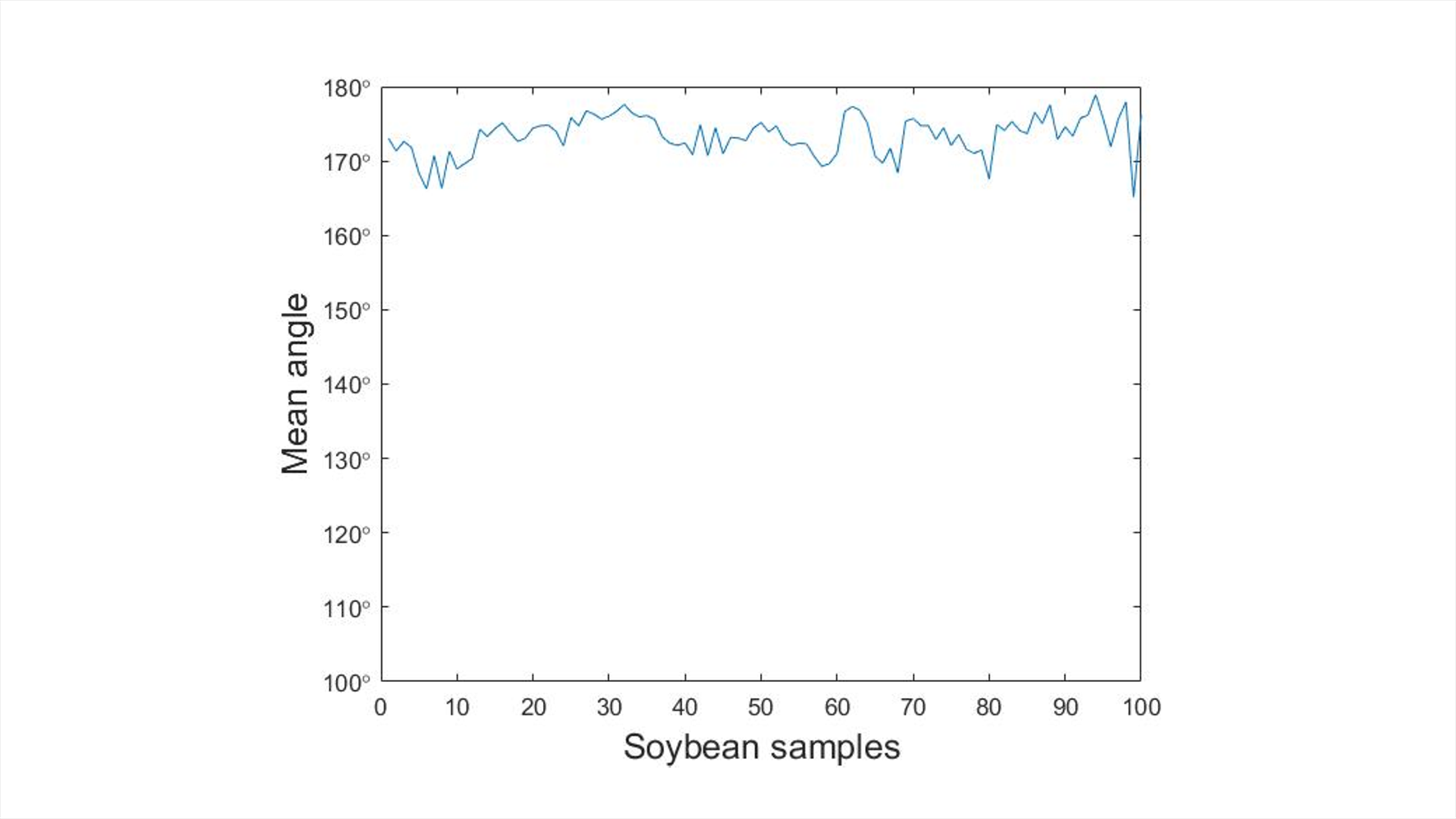

Supplement: Supplementary Figure 1 — Angle analysis of three adjacent stem nodes on branches of soybean plants. [file Image_1.TIFF]

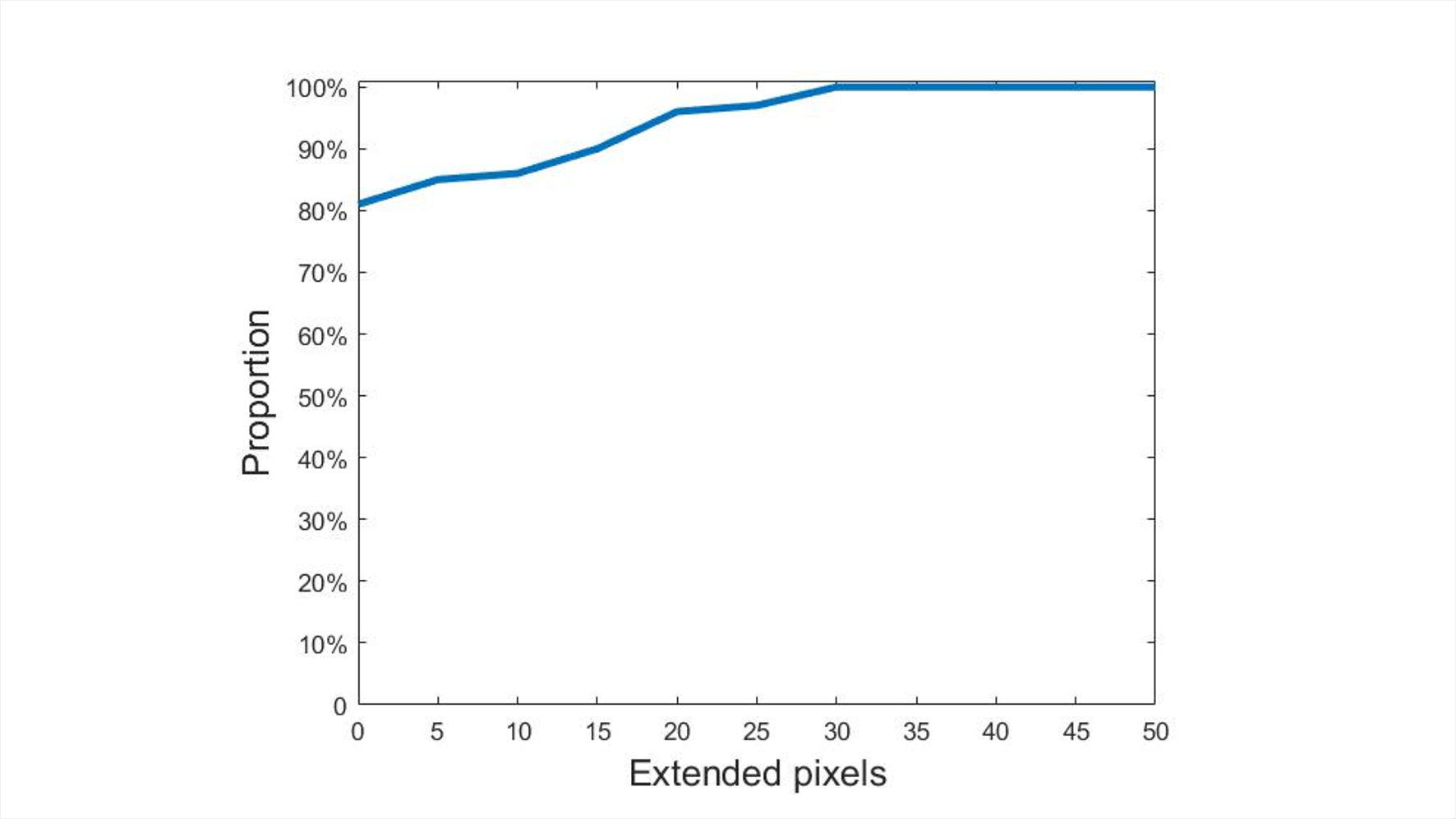

Supplement: Supplementary Figure 2 — Rectangular selection area extended pixel selection. [file Image_2.TIFF]
